# Supplementary material for: MetaQTL: a package of new computational methods for the meta-analysis of QTL mapping experiments
Source: BMC Bioinformatics. 2007 Feb 8;8:49. doi: 10.1186/1471-2105-8-49 (PMC1808479; doi:10.1186/1471-2105-8-49)
Supplement: Additional File 7 — MetaQTL Package : jar file and tutorial. This Zip archive contains both the MetaQTL JAR file and the files of the tutorial. [file 1471-2105-8-49-S7.zip › org.inra.metaqtl/doc/org/metaqtl/algo/ClustAlgorithm.html]

ClustAlgorithm


|  |  |  |
| --- | --- | --- |
| **Overview** \| **Package** \| **Class** \| **Use** \| **Tree** \| **Deprecated** \| **Index** \| **Help** | |  |
| PREV CLASS   **NEXT CLASS** | **FRAMES**    **NO FRAMES**     **All Classes** |  |
| SUMMARY: NESTED \| FIELD \| CONSTR \| METHOD | DETAIL: FIELD \| CONSTR \| METHOD |  |


---


## org.metaqtl.algo Class ClustAlgorithm

```
java.lang.Object
  org.metaqtl.algo.MetaAlgorithm
      org.metaqtl.algo.ClustAlgorithm
```

**All Implemented Interfaces:**: IMetaAlgorithm

**Direct Known Subclasses:**: QtlClustAlgorithm, QtlTreeAlgorithm

---

``` public abstract class ClustAlgorithm extends MetaAlgorithm ```

---

| **Field Summary** | |
| --- | --- |
| `protected  java.lang.String` | `chrName`             The name of the chromosome to study. |
| `protected  Chromosome[]` | `chromosomes`             The chromosome on which the clustering is performed. |
| `protected  int` | `missing_sd_mode`             This global variable defines the mode of imputation for the QTL for which the standard deviation of the position is not available. |
| `protected  IBioOntology` | `ontology`             The optional trait ontology to use to group Qtl. |
| `protected  MetaQtlAnalysis` | `result`             The result of the clustering. |
| `protected  int` | `sd_mode`             This global variable defines the mode of compuation of the standard deviation of the QTL positions. |

**Fields inherited from class org.metaqtl.algo.MetaAlgorithm**

`logger, loggerUp, workAmount, workProgress`


**Constructor Summary**

`ClustAlgorithm(IBioGenome map,
IBioOntology ontology)`

`ClustAlgorithm(IBioGenome map,
java.lang.String name,
IBioOntology ontology)`


| **Method Summary** | |
| --- | --- |
| `MetaQtlAnalysis` | `getResult()`             When the algorithm is finished use this method to get the result. |
| `void` | `setCIMiss(int missing_sd_mode)` |
| `void` | `setCIMode(int sd_mode)` |

**Methods inherited from class org.metaqtl.algo.MetaAlgorithm**

`disableLogger, getLogger, getWorkAmount, getWorkProgress, isLoggerEnable, setLogger, setLogger`

**Methods inherited from class java.lang.Object**

`clone, equals, finalize, getClass, hashCode, notify, notifyAll, toString, wait, wait, wait`

**Methods inherited from interface org.metaqtl.IMetaAlgorithm**

`run`

**Field Detail**

### result

```
protected MetaQtlAnalysis result
```

:   The result of the clustering.

---


### chromosomes

```
protected Chromosome[] chromosomes
```

:   The chromosome on which the clustering is performed.

---


### ontology

```
protected IBioOntology ontology
```

:   The optional trait ontology to use to group Qtl.

---


### chrName

```
protected java.lang.String chrName
```

:   The name of the chromosome to study.

---


### sd\_mode

```
protected int sd_mode
```

:   This global variable defines the mode of compuation of
    the standard deviation of the QTL positions.

---


### missing\_sd\_mode

```
protected int missing_sd_mode
```

:   This global variable defines the mode of imputation
    for the QTL for which the standard deviation of the position
    is not available.


**Constructor Detail**

### ClustAlgorithm

```
public ClustAlgorithm(IBioGenome map,
                      IBioOntology ontology)
```

**Parameters:**: `map` -: `ontology` -

---


### ClustAlgorithm

```
public ClustAlgorithm(IBioGenome map,
                      java.lang.String name,
                      IBioOntology ontology)
```


**Method Detail**

### setCIMode

```
public void setCIMode(int sd_mode)
```

:   **Parameters:**: `sd_mode` -

---


### setCIMiss

```
public void setCIMiss(int missing_sd_mode)
```

:   **Parameters:**: `missing_sd_mode` -

---


### getResult

```
public MetaQtlAnalysis getResult()
```

:   When the algorithm is finished use this method to get
    the result. It returns a `MetaQtlAnalysis` instance
    which store in each chromosome the different clustering models
    which have been tested.

    :   **Returns:**: a instance of `MetaQtlAnalysis`. **See Also:**: `MetaQtlAnalysis`


---


|  |  |  |
| --- | --- | --- |
| **Overview** \| **Package** \| **Class** \| **Use** \| **Tree** \| **Deprecated** \| **Index** \| **Help** | |  |
| PREV CLASS   **NEXT CLASS** | **FRAMES**    **NO FRAMES**     **All Classes** |  |
| SUMMARY: NESTED \| FIELD \| CONSTR \| METHOD | DETAIL: FIELD \| CONSTR \| METHOD |  |


---
